# The International Text-Book of Surgery, by British and American Authors

**Published:** 1903-03

**Authors:** 


**The International Text-book of Surgery, by British and American Authors.** Edited by A. Pearce Gould and J. Collins Warren. Vol. I. Pp. 947. London: W. B. Saunders & Co. 1900.

This book is one of the signs of the times—an Anglo-American combine of surgical literature—and the editors and contributors are to be congratulated on the thoroughness and completeness of their work.

This volume deals with general surgery, and the majority of the writers are well-known American surgeons. The orthography is transatlantic, as exemplified by such words as neighboring, oxid, chlorid, findings, theaters, tumors, and the composition is superior to that generally met with in American writings. The illustrations number over 450, and are of unequal merit, but include many excellent photographic reproductions. Special attention may be directed to a short but good chapter on the Surgical Pathology of the Blood; but we question the wisdom of the inclusion of a chapter on anæsthetics. The text is full of valuable detail, comprehensive and exact; and the work can be strongly recommended to surgical readers, and not less so from the fact that it expresses the views and practice of our kinsmen, which differ, in some respects, from our own.

